# Attenuation of Rhes Activity Significantly Delays the Appearance of Behavioral Symptoms in a Mouse Model of Huntington's Disease

**DOI:** 10.1371/journal.pone.0053606

**Published:** 2013-01-21

**Authors:** Brandon A. Baiamonte, Franklin A. Lee, Steve T. Brewer, Daniela Spano, Gerald J. LaHoste

**Affiliations:** 1 Applied Biopsychology Program, Department of Psychology, University of New Orleans, New Orleans, Louisiana, United States of America; 2 Dipartimento di Biochemica e Biotecnologie Mediche, Università di Napoli, Naples, Italy; 3 CEINGE Biotecnologie Avanzate, Naples, Italy; University G. D'Annunzio, Italy

## Abstract

Huntington's disease (HD) is a neuropsychiatric disorder characterized by choreiform movement of the limbs, cognitive disability, psychosis and dementia. It is invariably associated with an abnormally long CAG expansion within the IT15 gene on human chromosome 4. Although the mutant huntingtin protein is ubiquitously expressed in HD patients, cellular degeneration occurs predominantly in neurons within the corpus striatum and cerebral cortex. The Ras homolog Rhes is expressed very selectively in the precise brain areas affected by HD. Recent *in vitro* work suggests that Rhes may be a co-factor with mutant huntingtin in cell death. The objective of the present study was to examine whether the inhibition of Rhes would attenuate or delay the symptoms of HD *in vivo*. We used a transgenic mouse model of HD crossed with Rhes knockout mice to show that the behavioral symptoms of HD are regulated by Rhes. HD^+^/Rhes^−/−^ mice showed significantly delayed expression of HD-like symptoms in this *in vivo* model. Drugs that block or inhibit the actions of Rhes may be useful as the first treatments for HD.

## Introduction

Huntington's disease (HD) is a complex, progressive neurodegenerative disorder that results in deficits of motor function, cognition, and mood [1]. It is incurable, untreatable, and fatal. HD is caused by a genetic defect that is characterized by an unstable expansion of a trinucleotide repeat (CAG, which codes for glutamine) within the IT15 gene on human chromosome 4 [2]. Individuals with ≤35 repeats do not develop the HD phenotype, whereas those with >35 repeats invariably develop HD, usually beginning in middle-age [3]. The mutant gene (mHtt) is transmitted in an autosomal dominant matter, such that, on average, 50% of the offspring of an affected heterozygous individual will have HD. The long stretch of glutamine amino acids present in the mHtt protein (huntingtin) is believed to cause neurodegeneration and the symptoms that constitute HD [4]–[8].

The exact mechanism underlying neurodegeneration in HD is a matter of some debate [9]. A multitude of mechanisms responsible for this neurodegeneration, such as activation of proteases, protein misfolding, inhibition of protein degradation, transcriptional dysregulation, synaptic dysfunction, mitochondrial dysfunction, and oxidative stress have been proposed [10]. One serious problem for current theories of HD is that mHtt is present in all cells, yet only neurons within the corpus striatum and cerebral cortex of the brain are targeted for degeneration [11], [12]. This could be due to the remarkable similarity between the pattern of degeneration and the distribution of the mRNA for Rhes, the Ras homolog enriched in the striatum [13], [14]. Based on recent *in vitro* data in cultured cells, Subramaniam et al. [15] suggest that Rhes protein is a co-factor in mHtt-induced neurodegeneration. The rationale underlying this argument is that Rhes inhibits the formation of huntingtin aggregates, which, paradoxically, are believed to have a neuroprotective effect by sequestering soluble, cellular mHtt associated with cytotoxicity [15]–[18].

The present study was undertaken to determine whether the inhibition of Rhes would attenuate or delay the symptoms of HD *in vivo*. We report that doubly transgenic mice with a human HD gene insertion and Rhes deletion showed significantly delayed expression of HD-like symptoms.

## Methods

Prompted by these cellular findings we hypothesized that living, behaving HD mice would not manifest the usual abnormal symptoms in the absence of Rhes. We tested this hypothesis by cross-breeding mice that are transgenic for the human mHtt allele with Rhes knockout (KO) mice generated by homologous recombination [19]. Breeding pairs of mice transgenic for the human mHtt allele were obtained from Jackson Laboratory, Bar Harbor, ME (B6.Cg-Tg(Hdexon1)61 GpB/J; stock # 006741). The most common line of transgenic mice, R6/2 are reproductively incapable of breeding the crucial genotypes necessary to test our hypothesis. We therefore used the R6/1 line which contains ∼115 CAG repeats and shows symptoms that develop in mid-life that are homologous to the motor symptoms of HD. In addition, R6/1 mice develop behavioral symptoms later than the R6/2 line. The slower progression and later onset allows for a detailed analysis of the progression of the disease. Hemizygous males were bred with non-carrier females. For simplicity, we will refer to the Htt haplotype as NC (non-carrier) and to the mHtt-positive haplotype as HD; the Rhes genotypes will be referred to as WT (wild-type), Het (heterozygous), and KO (knockout). The crucial genotype (HD/KO) cannot be generated by simple cross-breeding. Thus, HD/KO mice were generated by secondary breeding of already cross-bred HD/Hets×HD/Hets. Six genotypes were tested: 1) NC/WT; 2) NC/Het; 3) NC/KO; 4) HD/WT; 5) HD/Het; and 6) HD/KO. We used both male and female mice with sample sizes of 7–8.

### Ethics Statement

All animals in the experiment were cared for in accordance with the guidelines of the U.S. Public Health Service Policy on Humane Care and Use of Laboratory Animals, and experimental protocols were approved and supervised by the University of New Orleans Institutional Animal Care and Use Committee (Protocol Numbers: UNO-09-011 and 09-012; Assurance Number: A3299-01).

### Statistics

The data were examined for statistical outliers and none were found. Therefore, no data were excluded from the analyses. All analyses were performed using SPSS for Windows (version 16.0) with the probability of a Type I error set at 0.05. Data for rotarod performance, limb adduction, brain weight, and brain area were analyzed in separate 2×3×6 mixed design factorial ANOVAs with HD gene (HD+, NC) and Rhes gene (WT, Het, KO) serving as between subjects variables and Testing Age (testing once every month) serving as a within-subjects variable. A 3-way interaction was followed by separate analyses of the HD gene×Rhes gene (2×2) interaction for each day of testing. Significant 2-way interactions on any day were followed by analysis of 1) the simple effects of HD on rotarod performance, limb adduction, brain weight, and brain area, 2) the simple effects of Rhes on rotarod performance, limb movements, brain weight, and brain area, and 3) interaction contrast to determine if the reduced amount of Rhes expression reversed or partially reversed the effects of HD on rotarod performance, limb movements, brain weight, and brain area. Tukey's HSD test was used for all post-hoc tests to control the family-wise error rate.

## Results and Discussion

### Motor coordination

**Figure 1:**
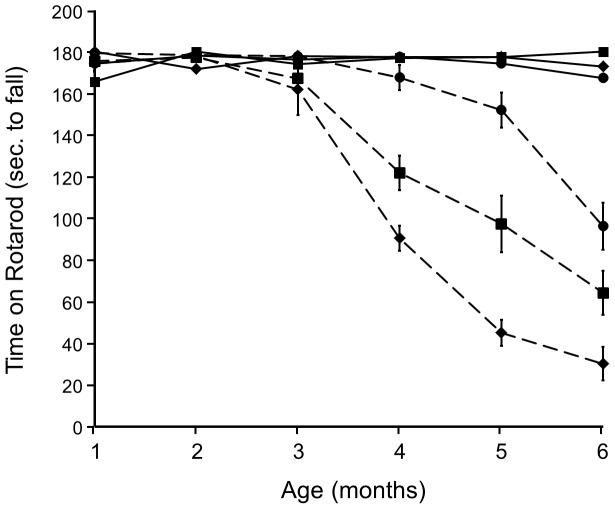
Rhes gene deletion significantly delays the onset of HD-induced motor dysfunction. NC: solid lines; HD: dashed lines; Diamonds: Rhes WT; Squares: Rhes Het; Circles: Rhes KO.

The mice were tested monthly from 1 to 6 months of age on a battery of previously validated motor behaviors [20]. Motor coordination was assessed using a circular, vertically rotating treadmill (Rotarod) at a fixed rotation speed of 16 rpm. Mice were placed on the treadmill with their noses opposite to the direction of rotation. The ability to stay on the treadmill without falling was taken as an index of motor coordination and balance. Three 60-sec. trials were given with 60-sec. intertrial intervals. All three groups of mice not carrying the HD allele (NC) performed perfectly well on the Rotarod test for the duration of the experiment (Fig. 1). In contrast, HD/WT mice showed a precipitous decline in motor coordination between months 3 and 4, worsening during the time-course of the study so that by 6 months of age the mean duration of time before falling was 20 sec. (max. duration, 180 sec.), representing an 88% decline in performance. However, HD/KO mice showed normal motor coordination until month 5 at which time their performance had declined significantly from NC mice, but only by 15%. Performance of HD/Het mice began to decline at 4 months of age, ultimately being significantly better than HD/WT and significantly worse than HD/KO mice, indicating a gene-dose effect. The HD mice differed as a function of Rhes genotype with respect to the age at which they showed ∼50% decline in motor performance. For the HD mice, we performed linear regression on data from months 3–6 to determine the age at which there was a 50% decline in motor performance. Analysis revealed these ages to be 4.33, 5.17, and 6.42 months. Thus, deletion of Rhes forestalled the symptoms of HD by 2.09 months. This amount of disease delay represents 6.5% of the lifespan of a control C57 mouse, or 25% of HD mice of this line [21], [22]. Although it is difficult to compare lifespans between species, it is likely that the delay in HD symptoms observed in the present experiment is clinically significant.

### Dystonia

**Figure 2:**
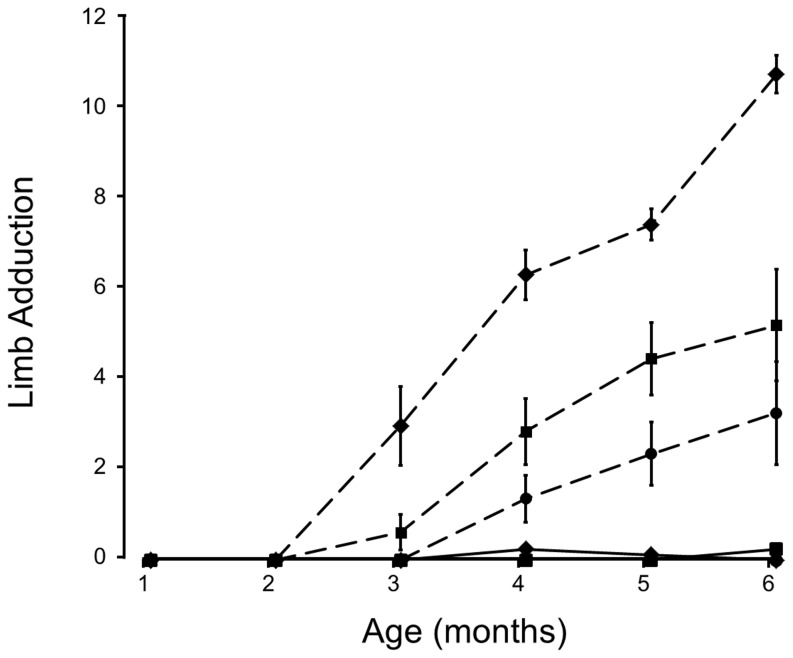
Dystonia as indicated by Limb Adduction. Legend is the same as for Fig. 1. Ordinate gives the average number of limbs drawn inward toward the body (labeled “Limb Adduction”) during three trials repeated monthly for the 6 months of observation.

When suspended from the tail normal mice splay their limbs outward; by contrast, mice with HD draw their limbs inward toward the body and clasp paired limbs together, an analogue of dystonia [22], [23]. Assessment of limb adduction entailed suspending each mouse by the tail and counting the number of limbs drawn inward during 3 monthly 10-sec trials [20]; clasping per se was not distinguished from adduction. The results were similar to those for the Rotarod test (Fig. 2). NC mice did not show limb adduction for the duration of the experiment. HD/WT mice showed significantly greater adduction than any other genotype from 3 months onward. HD/KO mice showed significantly less limb withdrawal than HD/WT or HD/Het mice during this period. HD/KO mice did not show significant limb adduction (relative to NC mice) until months 5 and 6. The amount of adduction for HD/Het mice was intermediate between HD/WT and HD/KO mice, further supporting a gene-dose effect.

### Brain measurements

**Figure 3:**
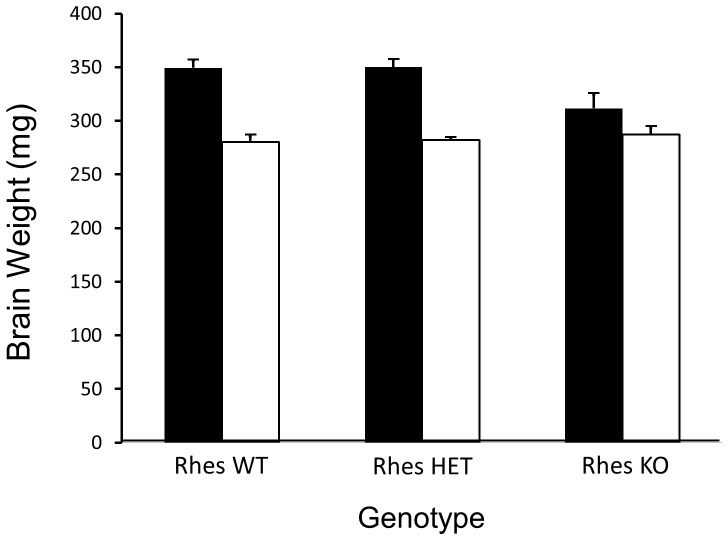
Post-mortem Brain Weight in mg. Black bars = NC; white bars = HD. Both Rhes and HD were significant factors in diminishing brain weight. However, a combined effect of HD and Rhes KO was not seen in HD/KO mice, indicating a protective effect of Rhes deletion on brain weight in these mice.

At the time of sacrifice, brains were flash-frozen in liquid isopentane (−20°C) on an analytical balance. As in clinical HD, and as previously show in mouse models, the brains of HD mice weighed significantly less (16%) than the brains of NC mice (Fig. 3). The brains of NC/KO mice weighed significantly less (11%) than those of other NC genotypes. In HD mice, there were no significant differences across the Rhes genotypes. However, there was a significant interaction between HD status and Rhes genotype such that the brain weights of NC/KO and HD/KO were not significantly different.

**Figure 4:**
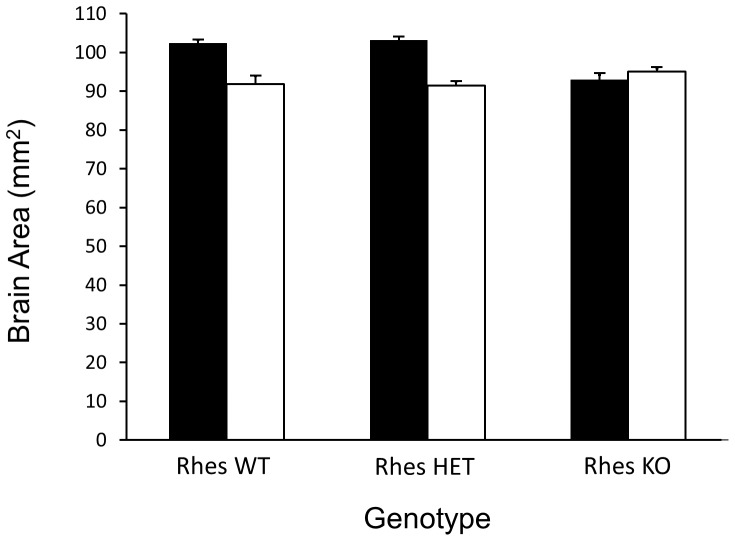
Brain Area (mm^2^) as a function of genotype. Legend is the same as in Fig. 3, and the conclusions are the same. Both Rhes and HD were significant factors in diminishing brain weight. However, a combined effect of HD and Rhes KO was not seen in HD/KO mice, indicating a protective effect of Rhes deletion on brain area loss in these mice.

With respect to a rudimentary measure of whole brain area, the same results were obtained as were found with brain weight. The dimensions of whole, frozen HD brains (measured as the product of antero-posterior distance of the cerebral cortex and the width of the brain at its widest point) were 7% less than the brains of NC mice. The dimensions of NC/KO mice were significantly less (10%) than those of other NC genotypes (Fig. 4). In HD mice, there were no significant differences across the Rhes genotypes. However, there was a significant interaction between HD status and Rhes genotype such that the brain weights of NC/KO and HD/KO were not significantly different. This pattern of results is identical to what was observed for brain weight.

### Body Weights

A 2×3 factorial Analysis of Variance showed that mice carrying the mHtt allele weighed significantly less (20%) than non-carrier mice. However, Rhes genotype was not a significant factor, nor was the interaction between Rhes genotype and HD genotype.

The present study shows that Rhes inhibition attenuates and delays the behavioral symptoms of HD *in vivo*. Our conclusion is based on the use of a Rhes knockout line that shows gene-dosage reduction (heterozygotes) and absence (knockouts) of Rhes protein (unpublished data). Our findings provide evidence of the ability of Rhes to augment HD-induced motor deficits. It is believed that Rhes does this by acting as an E3 ligase in the protein modification process called sumoylation. Subramaniam et al. [15], [24] have shown *in vitro* that Rhes interacts with the small ubiquitin-like modifier SUMO, which targets mHtt for sumoylation. In fact, Rhes increases sumoylation by 400% and is the principal determinant of sumoylation in the striatum [18], [24]. Increased binding of SUMO to mHtt to the exclusion of ubiquitin could lead to build-up of toxic protein.

Although previous findings suggested that SUMO indeed competed for ubiquitin at the same lysine [18], [25]–[27], it is now known that sumoylation can activate ubiquitination and increase protein clearance [28]. Thus, the interactions among Rhes, SUMOylation and mHtt may not be so simple. Steffan (2010) has proposed that SUMOylation, which can be induced by Rhes, may have an early protective effect in pre-symptomatic HD until age-related declines in protein clearing processes give rise to neurodegeneration and the appearance of symptoms. According to this thesis, Rhes might be expected to have a protective effect in the early, pre-symptomatic stages of HD. While this may be true in human HD, one would predict from this that in our mouse paradigm, HD/KO mice would show an earlier onset of *behavioral* symptoms compared to their HD/Rhes WT littermates, which they did not. We cannot rule out, however, a dual action of Rhes in neurodegeneration *per se*. An additional issue to be raised is that EGFP, contained in the Rhes knockout allele, and thereby confounded with Rhes genotype [19], may influence protein clearance processes [29].

In conclusion, the present findings show that inhibition of Rhes function by means of gene deletion delays the onset of behavioral symptoms in an animal model of HD, thereby identifying Rhes as a clinically significant target for the development of novel therapeutic strategies to treat the symptoms of HD.
